# LOXL1 and LOXL4 are novel target genes of the Zn^2+^-bound form of ZEB1 and play a crucial role in the acceleration of invasive events in triple-negative breast cancer cells

**DOI:** 10.3389/fonc.2023.1142886

**Published:** 2023-02-23

**Authors:** Daisuke Hirabayashi, Ken-ichi Yamamoto, Akihiro Maruyama, Nahoko Tomonobu, Rie Kinoshita, Youyi Chen, Ni Luh Gede Yoni Komalasari, Hitoshi Murata, Yuma Gohara, Fan Jiang, Jin Zhou, I Made Winarsa Ruma, I Wayan Sumardika, Akira Yamauchi, Futoshi Kuribayashi, Shinichi Toyooka, Yusuke Inoue, Masakiyo Sakaguchi

**Affiliations:** ^1^ Department of Cell Biology, Okayama University Graduate School of Medicine, Dentistry and Pharmaceutical Sciences, Okayama, Japan; ^2^ Department of General Surgery & Bio-Bank of General Surgery, The Fourth Affiliated Hospital of Harbin Medical University, Harbin, Heilongjiang, China; ^3^ Faculty of Medicine, Udayana University, Denpasar, Bali, Indonesia; ^4^ Medical Oncology Department of Gastrointestinal Tumors, Liaoning Cancer Hospital & Institute, Cancer Hospital of the Dalian University of Technology, Shenyang, Liaoning, China; ^5^ Department of Biochemistry, Kawasaki Medical School, Kurashiki, Okayama, Japan; ^6^ Department of General Thoracic Surgery and Breast and Endocrinological Surgery, Okayama University Graduate School of Medicine, Dentistry and Pharmaceutical Sciences, Okayama, Japan; ^7^ Faculty of Science and Technology, Division of Molecular Science, Gunma University, Kiryu, Gunma, Japan

**Keywords:** epithelial-to-mesenchymal transition, triple-negative breast cancer, zinc, ZEB1, metastasis

## Abstract

**Background:**

EMT has been proposed to be a crucial early event in cancer metastasis. EMT is rigidly regulated by the action of several EMT-core transcription factors, particularly ZEB1. We previously revealed an unusual role of ZEB1 in the S100A8/A9-mediated metastasis in breast cancer cells that expressed ZEB1 at a significant level and showed that the ZEB1 was activated on the MCAM-downstream pathway upon S100A8/A9 binding. ZEB1 is well known to require Zn^2+^ for its activation based on the presence of several Zn-finger motifs in the transcription factor. However, how Zn^2+^-binding works on the pleiotropic role of ZEB1 through cancer progression has not been fully elucidated.

**Methods:**

We established the engineered cells, MDA-MB-231 MutZEB1 (MDA-MutZEB1), that stably express MutZEB1 (ΔZn). The cells were then evaluated *in vitro* for their invasion activities. Finally, an RNA-Seq analysis was performed to compare the gene alteration profiles of the established cells comprehensively.

**Results:**

MDA-MutZEB1 showed a significant loss of the EMT, ultimately stalling the invasion. Inclusive analysis of the transcription changes after the expression of MutZEB1 (ΔZn) in MDA-MB-231 cells revealed the significant downregulation of LOX family genes, which are known to play a critical role in cancer metastasis. We found that LOXL1 and LOXL4 remarkably enhanced cancer invasiveness among the LOX family genes with altered expression.

**Conclusions:**

These findings indicate that ZEB1 potentiates Zn^2+^-mediated transcription of plural EMT-relevant factors, including LOXL1 and LOXL4, whose upregulation plays a critical role in the invasive dissemination of breast cancer cells.

## 1 Introduction

Through molecular-level analysis of the complex multistep process of metastasis, vulnerabilities that could be exploited to effectively prevent metastasis are being exposed. S100A8/A9, a heterodimer complex of S100A8 and S100A9, is an attractive molecule in this context because it becomes closely involved in cancer metastasis upon its abundant expression in the extracellular space (1). S100A8/A9 can stimulate toll-like receptor 4 (TLR4) (2), receptor for advanced glycation end products (RAGE), extracellular matrix metalloproteinase inducer (EMMPRIN) (3), melanoma cell adhesion molecule (MCAM) (4, 5), and neuroplastin (NPTN)β (6), which we collectively named the S100 soil sensor receptors (SSSRs) (7), resulting in the acquisition of metastatic force in several cancer cell lines. The expression of these receptors differs among cancer types and malignant stages (8). We previously reported that one of the SSSRs, i.e., MCAM, plays a notable role in S100A8/A9 binding in the context of metastatic outgrowth of triple-negative breast cancer cells (TNBCs) (9). Downstream of MCAM, we revealed that ETS translocation variant 4 (ETV4), also known as polyoma enhancer activator 3 (PEA3), is a key transcription factor (TF) for inducing metastasis of TNBCs through significant induction of zinc finger E-box binding homeobox 1 (ZEB1), a major epithelial-mesenchymal-transition (EMT) inducer. Therefore, we concluded that the ZEB1-mediated EMT empowers TNBCs to metastasize in response to S100A8/A9-MCAM signals (9).

ZEB1 is highly expressed in TNBCs and generally functions as a transcription repressor, although in some cases, it also acts as a transcription activator (10). The inverse transcription event will be regulated by multiple ZEB1-binding partners based on some post-translational modifications, such as phosphorylation and zinc ion (Zn^2+^)-binding status of ZEB1 (11–13). This event is attributed to the enriched Zn-finger motifs in the ZEB1 protein composition (14), but it has not been fully elucidated how Zn^2+^-binding influences the pleiotropic role of ZEB1 through cancer progression. Based on our experience and an extensive PubMed search, most of the published studies on ZEB1 involved experiments exploiting Zn^2+^-chelator (15), siRNA-based ZEB1 knock-down (10), and CRISPR/Cas9-mediated ZEB1 gene ablation (16, 17) methods, none of which can fully elucidate the Zn^2+^-mediated ZEB1 functions. In the present study, to fully address this subject, we produced a mutant ZEB1 expression construct (MutZEB1 (ΔZn): MutZEB1 for short) that results in a complete loss of the Zn^2+^-binding capacity of ZEB1 by using a novel approach, i.e., replacing all key amino acids that are essential to capture Zn^2+^ in all the Zn-finger motifs. By utilizing the new construct, we studied the role of Zn^2+^-binding by ZEB1 in the cellular behaviors of TNBCs *in vitro* in the context of cancer metastatic outgrowth. In this way, we sought to uncover the role of the Zn^2+^-possessing Zn-finger of ZEB1 on the dissemination of TNCBs *in vitro*.

## 2 Materials and methods

### 2.1 Cells

HEK293T cells, a non-cancerous human embryonic kidney epithelial cell line with stable expression of the simian virus 40 (SV40) large T antigen, were obtained from the RIKEN BioResource Center (Tsukuba, Japan). The human triple-negative breast cancer cell line MDA-MB-231 was obtained from ATCC (Rockville, MD). These cell lines were cultivated in DMEM/F12 medium (Thermo Fisher Scientific, Waltham, MA) supplemented with 10% FBS under a humidified incubator with 5% CO_2_ at 37°C.

### 2.2 MutZEB1

The codons corresponding to the amino acids cysteine and histidine in the Zn-finger motifs of ZEB1 were all replaced with serine. After that, all codons were optimized for human expression. The designed sequence was synthesized by FASMAC (Kanagawa, Japan), resulting in an artificial mutant cDNA of ZEB1, which we named MutZEB1 (ΔZn): MutZEB1 for short.

### 2.3 Plasmids

The mammalian gene expression constructs used in this study were all made using the pIDT-SMART -C-TSC vector (pCMViR-TSC) as the backbone to express the cargo genes. The plasmid vector has our original C-TSC cassette (C: CMV-RU5’ promoter located upstream of the cDNA; TSC: another promoter unit composed of triple tandem promoters, hTERT, SV40, and CMV, located downstream of the cDNA plus a polyadenylation signal), by which the developed plasmid shows far higher efficiency than that of potent conventional vector systems (18). The cDNAs located on the multi-cloning site of the pCMViR-TSC were all designed to be expressed in a C-terminal 3Flag-6His-tagged, 3Myc-6His-tagged or 3HA-6His-tagged form. The cDNAs encoding GFP, ZEB1 (WT), MutZEB1, and CTBP1 were inserted into the multi-cloning site of the pCMViR-TSC. Transient transfection of these plasmids into cultured cells was performed using FuGENE-HD (Promega BioSciences, San Luis Obispo, CA).

We used another of our original vectors, pSAKA-4B (9), to obtain stable transformants. cDNAs encoding GFP, MutZEB1, LOX, LOXL1, LOXL2, LOXL3, and LOXL4 were inserted into the multi-cloning site of pSAKA-4B. The clones stably overexpressing these genes were established by a convenient electroporation gene delivery method using Neon Transfection System (Thermo Fisher Scientific) and subsequent selection with puromycin at 20 µg/ml for one month.

### 2.4 Cell assays

#### 2.4.1 Proliferation

Cells (1 × 10^3^ cells) were seeded in culture plates (96-well plates). A CellTiter 96^®^ AQueous One Solution Cell Proliferation Assay (MTS) (Promega Biosciences) was used. The MTS values were presented as the average from three independent experiments.

#### 2.4.2 Colony formation

Cells (1 × 10^2^ cells) were seeded in a culture dish with a 6 cm diameter. On the 7th day, the formed cell colonies were fixed with 100% EtOH and stained with 1% crystal violet solution (Sigma-Aldrich, St. Lois, MO). The colonies composed of more than 20 cells were counted, and the number of colonies formed was presented as the average from three independent experiments.

#### 2.4.3 Cell attachment

Cells (1 × 10^4^ cells) were seeded in culture plates (96-well plates). After 30 min of incubation, non-attached cells were removed by washing with PBS. The attached cells were fixed with 100% EtOH, stained with 1% crystal violet solution (Sigma-Aldrich), and counted. Three independent experiments presented the number of stuck cells as the average.

#### 2.4.4 Migration and invasion

*In vitro* cell migration and invasion were evaluated by a Boyden chamber assay with a Matrigel-coated (for invasion) or non-coated (for migration) transwell membrane filter insert (pore size, 8 μm) in a 24-well plate (BD Biosciences, Franklin Lakes, NJ). Cells (2×10^4^ cells/insert) were seeded with low-serum (0.5% FBS) DMEM/F12 medium on the top chamber, and the bottom section was filled with DMEM/F12 medium containing 10% FBS. After incubation for 24 h, cells that passed through the filter were counted by staining with hematoxylin and eosin (H&E) solution. Migrated/invaded cells were imaged under a microscope (BZ-9000; KEYENCE, Tokyo) and quantified by cell counting in five non-overlapping fields at ×100 magnification. The numbers of migrated/invaded cells are the average from three independent experiments.

### 2.5 Real-time quantitative PCR

Total RNA was extracted from the cultured cells using RNeasy Mini Kit (Qiagen, Venlo, Netherlands). Reverse transcription was then performed using ReverTraAce qPCR RT Master Mix with gDNA Remover (Toyobo, Osaka, Japan). A real-time polymerase chain reaction (PCR) was performed on a LightCycler 480 system II (Roche Applied Science, Penzberg, Germany) for all gene transcripts of our interest according to the thermal profile set in **Table 1** using FastStart SYBR^®^ Green Master Mix (Roche Applied Science) with specific primers (**Table 2**) on a StepOnePlus™ Realtime PCR system (Applied Biosystems, Foster City, CA). The expression levels were normalized relative to *TBP* mRNA as an internal control using the ΔΔCt method. The RT-qPCR was repeated three times for each set of samples.

**Table 1:** Thermal profile.

Procedure	Temperature	Time
Hot start	95 °C	1 minute
× 40	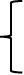 95 °C 60 °C	15 seconds
		30 seconds

**Table 2:** Primers.

Target	Forward (5’ to 3’)	Tm	Reverse (5’ to 3’)	Tm	Product (bp)
*TBP*	GAACATCATGGATCAGAACAACA	63.62	ATAGGGATTCCGGGAGT	63.28	87
*ZEB1*	GGAGGATGACACAGGAAAGG	63.45	TCTGCATCTGACTCGCATTC	64.22	105
*LOX*	TGAAAAACCAAGGGACATCAG	63.69	GGCATCAAGCAGGTCATAG	60.82	122
*LOXL1*	ACCAGGGCACAGCAGACTT	64.94	GTGGCTGCATCCAGTAGGTC	64.66	121
*LOXL2*	CTACGTGGAGGCCAAGTCC	64.61	CGTTGCCAGTACAGTGGAGA	63.94	92
*LOXL3*	CAACAGGAGGTTTGAACGCTAC	65.18	GCTGACATGGGTTTCTTGGTAA	65.84	118
*LOXL4*	TGCCGCTGCAAGTATGATG	66.01	TGTTCCTGAGACGCTGTTCC	65.42	112

### 2.6 Pull-down and immunoprecipitation

A pull-down experiment was performed using Zn-Nitrilotriacetic Acid (NTA) agarose beads (Cube Biotech, Monheim, Germany) or streptavidin-agarose beads (Thermo Fisher Scientific) coupled with the biotin-labeled ZEB1-responsive element oligo DNA that was synthesized by Sigma-Aldrich for the pull-down experiments. IP of the expressed foreign proteins was performed using anti-HA tag antibody-conjugated agarose beads (Sigma-Aldrich). The pull-down and IP experiments were repeated at least three times.

### 2.7 Western blotting

WB analysis was performed under conventional conditions. In brief, cell lysates were prepared using M-PER cell lysis buffer (Thermo Fisher Scientific). They were supplemented with SDS-sample buffer and subjected to electrophoresis on SDS polyacrylamide gel electrophoresis (SDS-PAGE) gel. The proteins separated according to their molecular masses were then transferred onto a polyvinylidene difluoride (PVDF) membrane (Thermo Fisher Scientific) using a semi-dry blotter (Nihoneido, Tokyo). The membrane was incubated with a blocking buffer (10% skim milk, 6% glycine, 0.1% Tween-20 in PBS) and then exposed to primary antibodies. The antibodies used were as follows: mouse anti-HA tag antibody (clone 6E2; Cell Signaling Technology, Danvers, MA), mouse anti-Myc tag antibody (clone 9B11; Cell Signaling Technology), mouse anti-Flag tag antibody (clone M2; Sigma-Aldrich), mouse anti-GFP antibody (Thermo Fisher Scientific), rabbit anti-ZEB1 antibody (Proteintech, Rosemont, IL), mouse anti-E-Cadherin antibody (Cell Signaling Technology), rabbit anti-N-Cadherin antibody (Cell Signaling Technology), mouse anti-Vimentin antibody (Agilent Technologies, Santa Clara, CA), rabbit anti-ZEB2 antibody (Proteintech), rabbit anti-Snail1 antibody (Cell Signaling Technology), and mouse anti-β-actin (Sigma-Aldrich) antibodies. The WB was repeated three times for each set of samples.

### 2.8 Immunocytochemistry

To visualize the proper expression of MutZEB1 tagged with Myc epitope at the C-terminal side in cells, the parental MDA-MB-231 cells and their MutZEB1 stable subline were fixed with 4% paraformaldehyde (PFA) and washed with 0.1% Triton X-100 in PBS. The expressed MutZEB1 protein was stained with mouse anti-Myc antibody (Cell Signaling Technology) as a primary antibody and further treated with Alexa Fluor 594–conjugated goat anti-mouse IgG antibody (Thermo Fisher Scientific) as a secondary antibody. Cell nuclei were counterstained with 4’,6-diamidino-2-phenylindole (DAPI). For the staining of actin filaments (F-actin), an F-actin probe (phalloidin) conjugated to tetramethylrhodamine (TRITC) was used (ActinRedTM 555 ReadyProbesTM reagent; Thermo Fisher Scientific).

### 2.9 RNA-Seq

According to the manufacturer’s instructions, total RNA was extracted from the cultured cells using RNeasy Mini Kit (Qiagen, Venlo, Netherlands). The RNA-Seq-based analysis of gene expression using the isolated RNA was consigned to the contract analysis service provided by Bioengineering Lab (Kanagawa, Japan). Sequencing libraries prepared by the company were loaded onto a DNBSEQ-G400 system (MGI Tech Japan, Hyogo, Japan). Trim Galore (version 0.6.4) with Cutadapt (version 2.7) was used to clean the reads and remove adapters. Filtered reads were mapped to the GENCODE Human Release 40 (GRCh38.p13) reference data using Salmon (version 1.6.0). For the DGE analyses, we used R packages (ver.4.1.0). Tximport (version 1.8.0) was used to import and summarize transcript-level abundance estimates for gene-level analysis with edgeR (version 3.34.1). DEG was defined as the gene with FDR less than 0.05.

### 2.10 Statistical analysis

Data are expressed as means ± standard deviation (SD). We used a simple pair-wise comparison with Student’s *t*-test (a two-tailed distribution with two-sample equal variance). Values of p<0.05 were considered statistically significant.

### 2.11 Data availability statement

The raw data supporting the conclusions of this article will be made available by the authors, without undue reservation. RNA-seq data that support the findings of this study have been deposited in the DDBJ Sequenced Read Archive under the accession numbers DRA015650 (https://www.ddbj.nig.ac.jp/dra/index.html).

## 3 Results

### 3.1 Construct of MutZEB1

**Figure 1:**
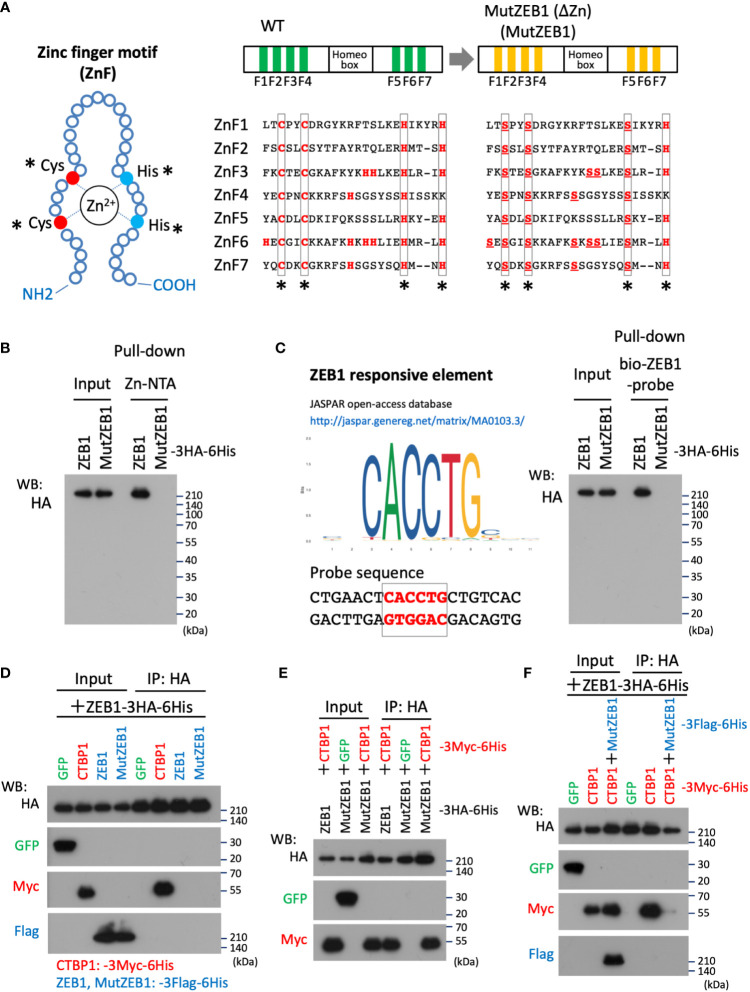
Design of the MutZEB1 construct and confirmation of its function. **(A)**, The composition of the zinc finger (ZnF) motif was depicted (left), in which the key amino acids, cysteine (Cys: C) and histidine (His: H), are essential to capture Zn^2+^ in the motif. In the motifs (ZnF1-7) of wild-type (WT) ZEB1, both C and H that are indicated with asterisks (*) were all replaced with serine (Ser: S) (right). To exclude the possibility of Zn^2+^-trapping by another H in the ZnFs, all the remaining H molecules were also replaced with S, resulting in a ZEB1 mutant construct that we named MutZEB1 (ΔZn): MutZEB1 for short (right). **(B)**, To confirm the loss of the Zn^2+^-binding ability of MutZEB1, HEK293T cells were transfected with HA-tagged ZEB1 (ZEB1 (WT)) or MutZEB1. Half of the total amount of transfected cell lysates was used as input to confirm the expression of the intracellularly delivered HA-tagged foreign products. The remaining half was subjected to a pull-down procedure using Zn-NTA agarose beads, which is helpful in collecting Zn^2+^-binding proteins selectively. After precipitation of the expressed products with the beads, bound foreign proteins were analyzed by western blotting using the HA antibody. **(C)**, To further confirm the loss of the DNA binding ability of MutZEB1, we performed a procedure similar to that described in the legend above **(B)**, except that streptavidin-beads coupled with a biotin-conjugated ZEB1-responsive element were used (left). After precipitation of the expressed products with the beads, bound foreign proteins were analyzed by western blotting with the HA antibody (right). **(D–F)**, The prepared cell lysates of HEK293T transfectants with different combinations of the plasmids, as indicated, were divided into two aliquots. The first was used as the whole cell lysate, i.e., as input, and the remaining half was used to conduct immunoprecipitations with anti-HA tag antibody-conjugated agarose beads. After the immunoprecipitation of the expressed products with the beads, bound foreign proteins were analyzed by western blotting with the indicated antibodies.

To develop Zn^2+^-binding-depleted ZEB1 protein, all Zn-finger motifs were mutated by replacing the key amino acid of cysteine or histidine, which is vital for capturing Zn^2+^ in the finger composition (**Figure 1A**, left) with serine, resulting in the construct MutZEB1 (**Figure 1A**, right). Pull-down assay of their force-expressed HEK293T cell lysates with Zn-NTA agarose beads confirmed that wild-type ZEB1 exhibited binding to Zn^2+^, but MutZEB1 did not (**Figure 1B**). We also found that MutZEB1 lost its DNA-binding ability, which was confirmed by a pull-down assay using the ZEB1-responsive consensus element (**Figure 1C**). To reveal the inhibitory role of MutZEB1 against the intrinsic wild-type of ZEB1 in cells, we first examined the potential of a dominant negative effect of MutZEB1 on the endogenous ZEB1. We examined whether MutZEB1 would bind with the wild-type of ZEB1 into an inactive ZEB1 homodimer. The binding experiment using the IP-WB technique with different combinations of the overexpressed-foreign proteins revealed no apparent homodimer formation between the wild-type ZEB1 and MutZEB1 (**Figure 1D**). In addition, no homodimerization of wild-type ZEB1 was observed, except for the positive binding with CTBP1, a renowned corepressor of ZEB1; this result has not been reported previously. However, CTBP1 binding occurred in MutZEB1 just as in the wild-type protein (**Figure 1E**). When MutZEB1 was further combined with wild-type ZEB1 and CTBP1, the binding of CTBP1 with the wild-type ZEB1 was markedly reduced (**Figure 1F**). These results indicate that MutZEB1 competes with wild-type ZEB1 for the ZEB1 target genes in transcriptional regulation through binding with plural ZEB1-binding partners but not those that show Zn^2+^-dependent binding.

### 3.2 Establishment of a subline of MutZEB1-overexpressing cells

**Figure 2:**
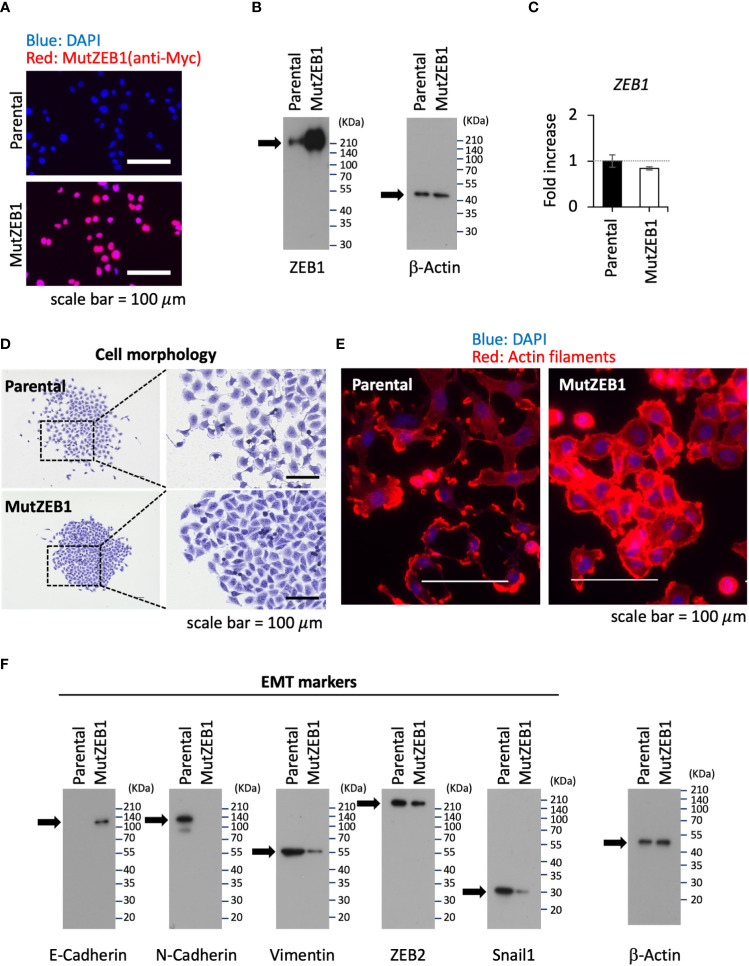
Inhibitory role of MutZEB1 on EMT. **(A)**, The established MDA-MB-231 subline that overexpresses MutZEB1 stably was immunofluorescence-stained by an anti-Myc tag antibody for the expressed Myc-tagged MutZEB1. The DAPI staining detected nuclei. The bars in the images represent 100 μm. **(B)**, The expressed MutZEB1 in the subline was also detected by an anti-ZEB1 antibody using the WB procedure. **(C)**, Real-time qPCR was performed to learn the endogenous level of *ZEB1* mRNA in the subline. The primer pair of ZEB1 could not be used to amplify the foreign *MutZEB1* because its codons through the coding region were optimized for human expression. *TBP* RNA was used as a control for the analysis. **(D, E)**, Cell morphology **(D)** and actin fibers **(E)** were observed for the subline in comparison to the parental MDA-MB-231 cells after staining them with the crystal violet dye **(D)** and F-actin probe (phalloidin) conjugated to tetramethylrhodamine (TRITC) **(E)**, respectively. The bars in the images represent 100 μm. **(F)**, The subline and its parental cells were subjected to WB analysis to detect representative EMT markers as indicated.

**Figure 3:**
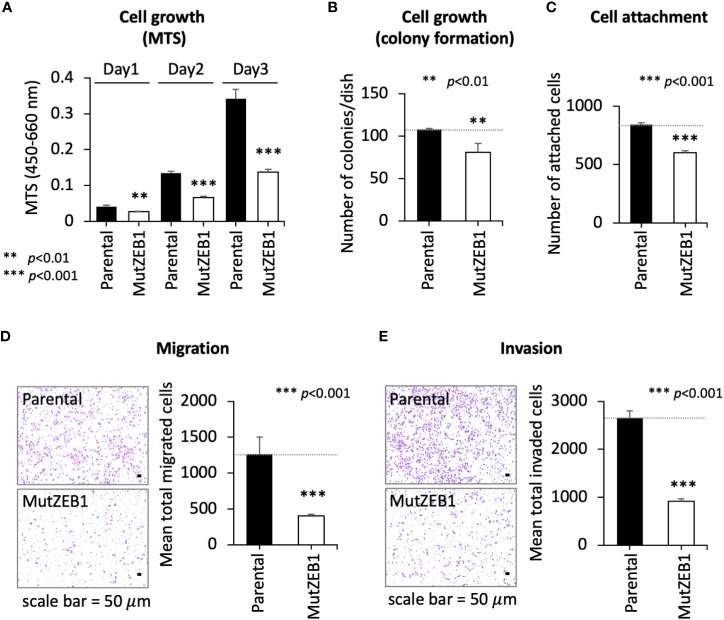
Inhibitory role of MutZEB1 on several cancerous cellular processes. **(A–E)**, The parental cells and their MutZEB1-expressing subline were evaluated for their cell growth **(A, B)**, cell attachment **(C)**, migration **(D)**, and invasion **(E)** by using MTS **(A)**, counting of colonies **(B)**, counting of the cells attached to the bottom of the culture dish **(C)**, and Boyden chamber assays (using a cell passing chamber membrane with no coating **(D)** or coated with Matrigel **(E)**). The bars in the migrated cell images represent 50 μm [images at left in panels **(D, E)**]. Data from **(A)** through **(E)** are means ± SD, **P < 0.01, and ***P < 0.001 by Student’s *t-*test.

Using the MutZEB1 construct, we next established cells overexpressing MutZEB1 from the MDA-MB-231 cell line. The resulting cells were designated MDA-MutZEB1 and showed a sustainable expression of the foreign MutZEB1. The expression of MutZEB1 in the established subline was confirmed by immunocytochemistry for the nuclear localization (**Figure 2A**) and by western blotting for the expressed protein level compared to the endogenous ZEB1 (**Figure 2B**). The expressed ectopic MutZEB1 did not affect the mRNA level of endogenous ZEB1 (**Figure 2C**). Owing to the nature of the MutZEB1 DNA sequence, whose codons were optimized mainly for human expression, the primer pair of wild-type ZEB1 was not suitable for detecting MutZEB1 in the real-time qPCR analysis. Intriguingly, MDA-MutZEB1 showed an epithelial shape and tighter cell–cell attachment than the parental cells, suggesting that MDA-MutZEB1 had undergone mesenchymal-to-epithelial transition (MET) (**Figure 2D**). The fibrous actin staining well mirrored the epithelial trait of MDA-MutZEB1—that is, the actin filaments were highly enriched at membrane rims where the cells were tightly tethered together (**Figure 2E**). The western blotting showed an increase in the level of a representative epithelial marker, E-cadherin, and the downregulation of several EMT markers, i.e., N-cadherin, vimentin, and snail1 (but not ZEB2) (**Figure 2F**). Enhancement of cancer cell growth, migration, and invasion activities can be caused by EMT events (19, 20). Therefore, we examined these cellular activities in MDA-MutZEB1. The standard cell growth assay based on the MTS assay showed a stalled proliferation activity in the subline compared to that of parental cells (**Figure 3A**). Similar to the results of the MTS assay, in the standard cell growth assay, colony formation was also dampened by the MutZEB1 expression (**Figure 3B**). In agreement with these findings, cell attachment (**Figure 3C**), migration (**Figure 3D**), and invasion (**Figure 3E**) were all significantly reduced in MDA-MutZEB1. These results indicate that MutZEB1 can function as a valuable tool to reverse EMT without affecting the endogenous levels of ZEB1 and ZEB2 since changes in ZEB1 and ZEB2 can weaken the EMT-linked cellular activities such as proliferation, attachment, migration, and invasion in TNBCs.

### 3.3 LOXL1 and LOXL4 are novel ZEB1 regulatory genes that are pioneer factors in cancer invasion

**Figure 4:**
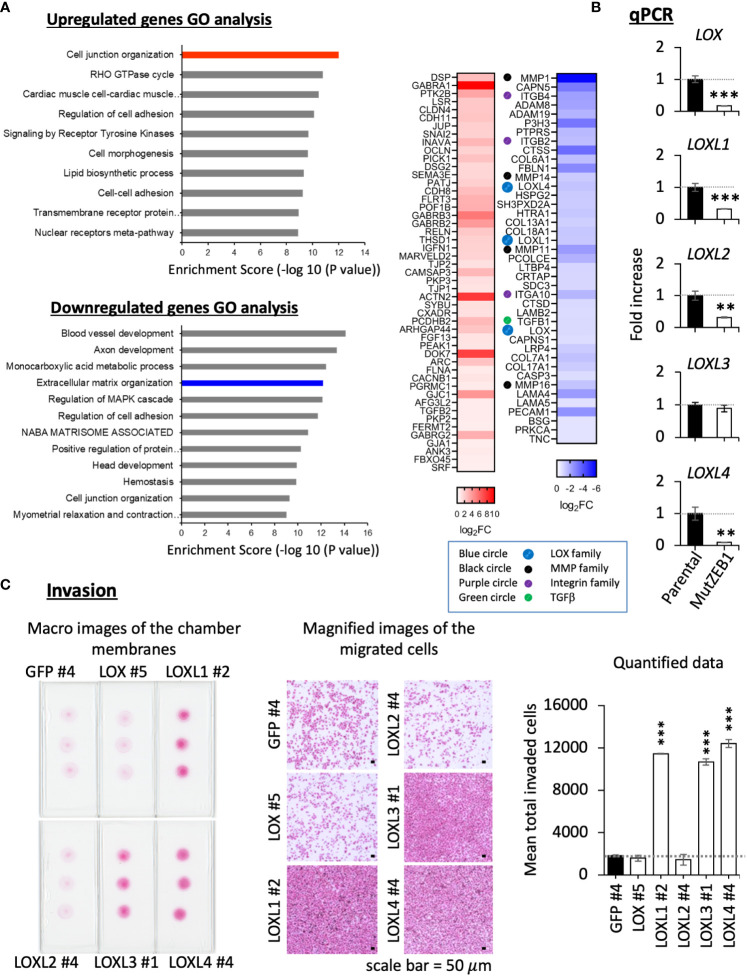
Effect of MutZEB1 on the ZEB1-downstream gene expressions. **(A)**, RNA-Seq analysis was done for the parental MDA-MB-231 cells and their MutZEB1-expressing subline. Gene ontology (GO) enrichment analysis (P<0.05) was performed for protein-coding RNAs to identify upregulated or downregulated genes in the subline in comparison to those in the parental cells (left). Heat maps were displayed for the selected upregulated and downregulated genes in the cell junction organization and extracellular matrix organization categories, respectively (right). These analyses were performed using the database for annotation, visualization, and integrated discovery (DAVID) v6.8 (http://david.ncifcr.gov/). In the heat map, the downregulated genes of our interest were highlighted by the colored circles (lysyl oxidases (LOXs): blue, matrix metalloproteases (MMPs): black, integrins (ITGs): purple, and TGFB1: green). **(B)**, Total RNAs prepared from the indicated cells (parental MDA-MB-231 cells and their MutZEB1-expressing subline) were analyzed for the expression of *LOX family* gene receptors (*LOX*, *LOXL1*, *LOXL2*, *LOXL3*, and *LOXL4*) by real-time qPCR. *TBP* mRNA was used as a control for the analysis. **(C)**, Invasion activities were evaluated in the individual LOX-overexpressing cell sublines employing a Boyden chamber-based invasion assay. The chamber membrane with the cells that had passed through the membrane was stained with hematoxylin-eosin (H&E) and placed on the slide glasses. The slide glasses were then observed macroscopically (left) and microscopically (middle) to count the number of stained cells (right). The bars in the migrated cell images represent 50 μm (middle). Data from **(B, C)** (right) are means ± SD, **P < 0.01, and ***P < 0.001 by Student’s *t-*test.

Using MDA-MutZEB1, we next comprehensively examined the changes in gene expression profiles to uncover novel ZEB1 regulatory genes that play an important role in the metastatic progression of TNBCs. The RNA-Seq data revealed global changes induced by the MutZEB1 expression. The gene ontology (GO) enrichment analysis (**Figure 4A**, left) announced that the biological functions of the upregulated genes of our interest are related to cell junction organization, the RHO GTPase cycle, regulation of cell adhesion, and cell–cell adhesion, which are all associated with EMT. The downregulated genes were also involved in interesting biological processes related to not only the regulation of cell adhesion and cell junction organization but also extracellular matrix organization, which is important for establishing the cancer metastatic milieu. Our attention was drawn to the results of the heat map readout of extracellular matrix organization in the downregulated genes rather than the heat map readout of cell junction organization in the upregulated genes (**Figure 4A**, right). Due to their solid digesting function, the matrix metalloproteases MMP1, MMP11, MMP14, and MMP16 might confer invasiveness to breast cancer cells through the Zn^2+^-bound ZEB1. Through the acquisition of adhesion ability, the integrins ITGB2, ITGB4, and ITGA10 may promote the attachment of breast cancer cells to the extracellular matrix and thereby enhance metastasis. In addition, TGFβ1 is well known to enhance EMT, cell migration, and extracellular matrix production. Moreover, the lysyl oxidase (LOX) family members LOX, LOXL1, and LOXL4 have attracted much attention due to their critical role in cancer metastasis. However, to our knowledge, there has yet to be a report about their regulation by ZEB1. In the present study, our real-time qPCR analysis confirmed that the expression of these LOX family members was reduced in MDA-MutZEB1 (**Figure 4B**), and we also found that the levels of LOX family genes except for LOXL3—that is, LOX, LOXL1, LOXL2, and LOXL4—were lowered by the presence of MutZEB1. Among the proteins encoded by these four genes, we found that LOXL1 and LOXL4 induced remarkable levels of invasiveness in MDA-MB-231 parental cells (**Figure 4C**) when we established MDA-MB-231-LOX sublines that stably overexpressed each of the corresponding LOX family proteins (**Supplementary Figure 1A**). In addition, LOXL4 but not LOXL1 overexpression was associated with a very high level of gelatin digestion (**Supplementary Figure 1B**), which may explain how ZEB1-mediated upregulation of MMPs is caused by ZEB1-induced LOXL4. Taken together, the results of our study reveal a critical role of Zn^2+^-bound ZEB1 in the progression of TNBCs through the regulation of multiple genes that include new ZEB1 target genes, LOXL1 and LOXL4.

## 4 Discussion

In the present study, we showed that our construct MutZEB1 inhibits the intrinsic wild-type ZEB1 in MDA-MB-231 cells, which effectively dampens EMT, migration, and invasion activities through the competitive binding of the transcriptional regulatory proteins of ZEB1 such as CTBP1. We further identified multiple cancer-relevant target genes of the Zn^2+^-activated ZEB1, among which LOXL1 and LOXL2, in particular, function as pioneer factors for invasiveness. Thus, the developed construct will continue to be a valuable tool for studying the Zn^2+^-bound ZEB1 processes revealed herein. Such future analysis should help untangle the complex mechanisms underlying the contradictory roles of ZEB1 as a transcriptional suppressor and transcriptional promoter in specific cellular contexts and cell types.

In general, it is common for transcription factors to function cooperatively with the proteins to which they are coupled. It has been reported that ZEB1 has multiple binding partners, which regulate the expression of many genes through cancer progression (21). For instance, ZEB1 interacts cooperatively with estrogen receptor α (ERα) in the early stage of breast cancer, and this interaction enhances the expression of ERα-mediated genes such as ANXA2 and CD151, which play a crucial role in EMT and subsequent bone tropic metastasis of early-stage breast cancer cells (22). In TNBCs that lack ERα, ZEB1 binds with YAP1, which functions as a transcriptional activator of several genes related to not only metastasis-relevant EMT but also stemness, proliferation, and chemoresistance (10). To interact with YAP1, ZEB1 may require the binding of Zn^2+^. Liu et al. reported that an intracellular increase in Zn^2+^ levels induced in part through the actions of the Zn^2+^-transporter ZIP4 resulted in the formation of the ZEB1/YAP1 complex, and thereby promoted EMT and metastasis of pancreatic cancer cells (14). A similar action would occur in TNBCs since ZIP4 is upregulated in TNBCs. In our attempt to comprehensively identify the ZEB1-interactive proteins whose interactions are regulated by the binding of Zn^2+^ to ZEB1 by mass spectrometric analysis, we failed in detecting YAP1, probably due to its low affinity with ZEB1. Still, we found an exciting transcription factor, ZEB2 (**Supplementary Figure 2A**). Surprisingly, this is the first report on the Zn^2+^-dependent interaction between ZEB1 and ZEB2. ZEB2 is a cognate transcription factor to ZEB1 and, like ZEB1, is a master EMT regulator highly expressed in TNBCs (23). Further analysis of the novel ZEB1 heterodimer complex with ZEB2 may open a pathway to elucidate the uncovered functions of ZEB1 in breast cancer progression. Interestingly, by mining our RNA-Seq data, we found that the Zn^2+^-bound ZEB1 activates multiple genes relevant to cancer metastasis, including the MMP family, integrin family, and LOX family genes. Among these genes, the LOX family genes are of particular interest since, although their functions in cancer metastasis are still mostly enigmatic, accumulating evidence shows that they play a significant role in the metastatic progression of breast cancer cells. LOX proteins generally mediate the lysyl oxidation of collagen, leading to cross-linked mature collagen through their catalytic activities. Therefore, LOXs have long been considered involved in cancer progression through cancer-stroma-matrix rearrangements suited to the metastatic outgrowth of cancer cells (24–26). However, very intriguingly, another function of LOX in cancer cells was also recently reported, which is not dependent on the collagen arrangements (27). Our present data indeed show that LOXL1 and LOXL4 are upregulated by the Zn^2+^-bound ZEB1 and promote invasion at exceptional levels in MDA-MB-231 cells. In addition, the overexpression of LOXL4 but not LOXL1 led to significant digestion of gelatin (**Supplementary Figure 1B**). Of our interest, the wild-type Zn^2+^-bound ZEB1 but not the Zn^2+^-unbound MutZEB1 was able to bind both LOXL1 and LOXL4 promoters (**Supplementary Figures 2B, C**). Conceivably, the two genes would be regulated by the Zn^2+^-mediated single ZEB1 or its heterodimer complex with other proteins, including ZEB2 we newly found, and contribute to activating the invasiveness of TNCBs. Plans to investigate this exciting possibility are currently underway at our laboratory.

In conclusion, the study of MutZEB1 revealed that Zn^2+^-binding is essential for the functions of ZEB1 in promoting EMT, migration, and invasion through multiple transcription regulations. The analysis of the altered gene expression profiles combined with cell-based invasion assays uncovered two new ZEB1 target molecules, LOXL1 and LOXL4, that play a crucial role in the manifestation of aggressive invasiveness of TNBCs. The Zn^2+^-dependent fashioning of ZEB1/ZEB2 may be involved in regulating the expression of these molecules. Our new approach utilizing MutZEB1 will be helpful in further elucidating the roles of ZEB1 in cancer biology.

## Data availability statement

The data presented in this study are deposited in the DDBJ Sequenced Read Archive repository (https://ddbj.nig.ac.jp/search), accession number DRA015650.

## Author contributions

DH designed, and DH and K-IY performed most of the experiments and analyzed the data. AM made a stable subline expressing MutZEB1 and performed several cell assays. NT provided advice and assistance with the cell assays. RK performed RNA-Seq analysis. YC made stable cell lines expressing the LOX family and performed an invasion assay. NK performed migration and invasion assays. HM, YG, FJ, JZ, IR, and IS helped confirm several *in vitro* results. AY, FK, and ST assisted with the data analysis. YI performed real-time qPCR analysis. MS performed the plasmid construction, immunoprecipitation, and Western blot analysis with the assistance of. DH and K-IY DH and MS wrote the manuscript. MS designed and supervised the project, and reviewed and edited the manuscript. All authors contributed to the article and approved the submitted version.
