# Supplementary material for: LOXL1 and LOXL4 are novel target genes of the Zn2+-bound form of ZEB1 and play a crucial role in the acceleration of invasive events in triple-negative breast cancer cells
Source: Front Oncol. 2023 Feb 23;13:1142886. doi: 10.3389/fonc.2023.1142886 (PMC9997211; doi:10.3389/fonc.2023.1142886)
Supplement: Supplementary file 1 [file DataSheet_1.docx]

Supplementary Material

LOXL1 and LOXL4 are novel target genes of the Zn^2+^-bound form of ZEB1 and play a crucial role in the acceleration of invasive events of triple-negative breast cancer cells

Daisuke Hirabayashi^1†^, Ken-ichi Yamamoto^1†^, Akihiro Maruyama^1^, Nahoko Tomonobu^1^, Rie Kinoshita^1^, Youyi Chen^2^, Ni Luh Gede Yoni Komalasari^1,3^, Hitoshi Murata^1^, Yuma Gohara^1^, Fan Jiang^1^, Jin Zhou^4^, I Made Winarsa Ruma^3^, I Wayan Sumardika^3^, Akira Yamauchi^5^, Futoshi Kuribayashi^5^, Shinichi Toyooka^6^, Yusuke Inoue^7^, Masakiyo Sakaguchi^1*^

^1^ Department of Cell Biology, Okayama University Graduate School of Medicine, Dentistry and Pharmaceutical Sciences, Okayama, Japan.

^2^ Department of General Surgery & Bio-Bank of General Surgery, The Fourth Affiliated Hospital of Harbin Medical University, Harbin, Heilongjiang, China.

^3^ Faculty of Medicine, Udayana University, Denpasar, Bali, Indonesia.

^4^ Medical Oncology Department of Gastrointestinal Tumors, Liaoning Cancer Hospital & Institute, Cancer Hospital of the Dalian University of Technology, Shenyang, Liaoning, China.

^5^ Department of Biochemistry, Kawasaki Medical School, Kurashiki, Okayama, Japan.

^6^ Department of General Thoracic Surgery and Breast and Endocrinological Surgery, Okayama University Graduate School of Medicine, Dentistry and Pharmaceutical Sciences, Okayama, Japan

^7^ Faculty of Science and Technology, Division of Molecular Science, Gunma University, Kiryu, Gunma, Japan.

^†^These authors contributed equally to this work and share the first authorship.

***Corresponding Author**: Masakiyo Sakaguchi, Ph.D.
E-mail: [masa-s@md.okayama-u.ac.jp](mailto:masa-s@md.okayama-u.ac.jp); Phone number: +81-86-235-7395; Fax: +81-86-235-7400; Department of Cell Biology, Okayama University Graduate School of Medicine, Dentistry and Pharmaceutical Sciences, 2-5-1 Shikata-cho, Kita-ku, Okayama-shi, Okayama 700-8558, Japan

# Supplementary Figures

## Supplementary Figure 1.


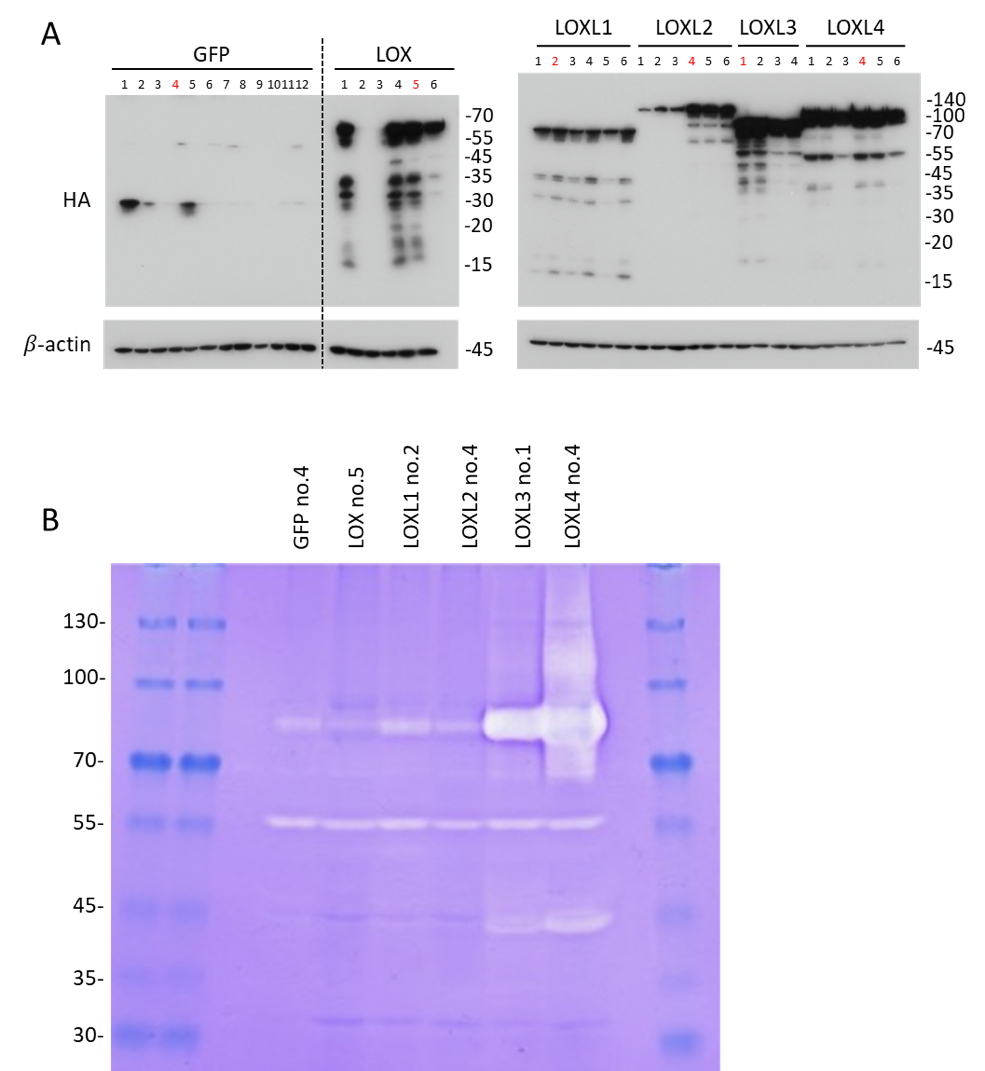


**Supplementary Figure 1. Establishment of MDA-MB-231-LOX sublines that stably overexpress individual LOX family proteins and evaluation of the activities of their secreted MMPs. A**, Establishment of MDA-MB-231-derived sublines that stably express foreign GFP, LOX, LOXL1, LOXL2, LOXL3, or LOXL4; all these foreign proteins were designed to have an HA tag at the C-terminal side. Selected clones resistant to puromycin selection were examined by WB for the transduced foreign genes, including the indicated genes using an anti-HA tag antibody. One of each of the clones that were positive for the transduced genes was randomly chosen (the selected clones are shown in red), resulting in the selection of GFP (#4), LOX (#5), LOXL1 (#2), LOXL2 (#4), LOXL3 (#1), and LOXL4 (#4) clones. **B**, Conditioned media were collected from individual cell sublines as indicated under serum-free cultivation for 24 h. Gelatin zymography was performed to assess the levels of matured MMPs in the indicated cell sublines. Equal amounts of the collected cell-conditioned media were separated by electrophoresis in polyacrylamide gel polymerized with 1% bovine gelatin. Three washes were made with 2.5% Triton and incubated for 24 h at 37 °C with MMPs activation buffer (50 mM Tris/pH 7.4 and 5 mM of CaCl_2_). The gels were stained with 0.1% Coomassie Brilliant Blue R-250 and de-stained with 10% acetic acid and 30% ethanol in water. The gelatinolytic activity was detected as unstained bands on a blue background.

## Supplementary Figure 2.

**Supplementary Figure 2. Identification of interacting proteins with ZEB1 and binding of ZEB1 to the LOXL1 and LOXL4 promoters. A**, Identification of the binding proteins for the ZEB1 (WT) or MutZEB1. HEK293T cells were transfected with GFP, HA-tagged ZEB1 (ZEB1 (WT)), or MutZEB1. The lysates of the three resulting cell lines were then subjected to an immunoprecipitation procedure using anti-HA-tag antibody-conjugated agarose beads. The collected immunoprecipitates were washed with PBS, and the bound proteins were eluted from the beads by an acidic buffer (100 mM of glycine-HCl buffer/pH 2.0), neutralized with 1.0 M of Tris-HCl buffer/pH 9.0, dialyzed with trypsin digestion buffer (10 mM CaCl2, 100 mM of ammonium bicarbonate/pH7.8), and trypsinized overnight at 37 °C. The digested proteins were directly subjected to a shotgun-type protein identification approach using a nano-flow liquid chromatography-mass spectrometry apparatus (Agilent 6330 Ion Trap; Agilent Technologies, Santa Clara, CA) equipped with an analytical chip (Agilent HPLC-Chip; Agilent Technologies). The resulting tandem mass spectrometry spectra of the tryptic peptides were finally analyzed using Agilent software (Spectrum Mill MS Proteomics Workbench; Agilent Technologies) and the SwissProt Homo sapiens protein database. The identified protein names in the control GFP transfectant were deleted from the lists of the ZEB1 (WT) and MutZEB1. The yellow and grey colored columns were specifically appeared proteins in ZEB1 (WT) and MutZEB1, respectively. **B**, **C**, ChIP-PCR of ZEB1 on the LOXL1 and LOXL4 promoters. The chromatin immunoprecipitation (ChIP) assay was performed in the gene-delivered HEK293T cells with the pCMViR-TSC-ZEB1 or -MutZEB1 (ΔZn) by using the rabbit anti-HA antibody (MBL, Nagoya, Japan) with the CUT & RUN assay kit (Cell Signaling Technology) according to the manufacturer’s direction. After purification of the digested DNA fragments from the cells, the target DNA fragment was amplified and determined by polymerase chain reaction. The amplified PCR products were separated by 4% polyacrylamide gel and then detected by the ethidium bromide (EtBr) staining. Primers were designed to enclose ZEB1 binding sites on the LOXL1 or LOXL4 promoter region. The used forward and reverse primer pairs (5′ to 3′) were as follows: pLOXL1-ZEB1 #1 (forward: CCATCTGGCCTAATCTTCCA; reverse: TGAACAACAGAAGGGGGTTC; Product Size: 116 bp), pLOXL1-ZEB1 #2 (forward: CAGCTCCAGTGCCTCTCTTC; reverse: ACACCCTCTTTGCATGTTCC; Product Size: 184 bp), pLOXL4-ZEB1 (forward: AACTGCCTGGGAGAGGGACT; reverse: CCCTGGGGCCTTTTTATCC; Product Size: 330 bp). We found two and one ZEB1 responsive elements, as shown in **Figure 1C**, in the LOXL1 promoter and LOXL4 promoter, respectively **(B**). According to the method mentioned above, we performed a CHIP assay for both LOXL1 and LOXL4 promoters, and we found that ZEB1 (WT) but not MutZEB1 can bind all the ZEB1 responsive elements in both LOXL1 and LOXL4 promoters.

**
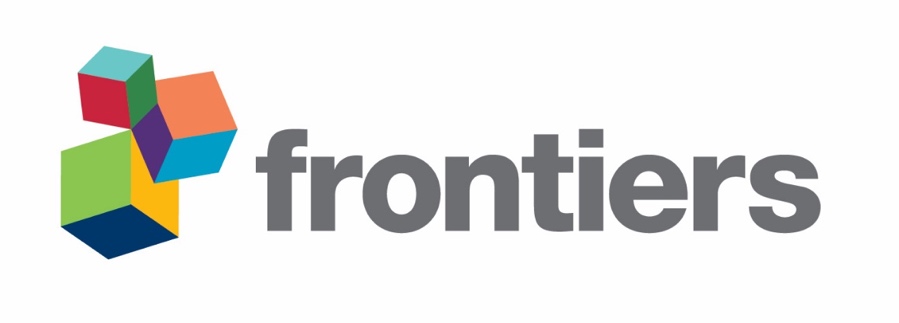
**
